# *N*,*N*′-Bis(2-methyl­phen­yl)succinamide

**DOI:** 10.1107/S1600536811004442

**Published:** 2011-02-12

**Authors:** B. S. Saraswathi, Sabine Foro, B. Thimme Gowda

**Affiliations:** a Department of Chemistry, Mangalore University, Mangalagangotri 574 199, Mangalore, India; b Institute of Materials Science, Darmstadt University of Technology, Petersenstrasse 23, D-64287 Darmstadt, Germany

## Abstract

In the title compound, C_18_H_20_N_2_O_2_, the conformations of the N—H and C=O bonds in the C—NH—C(O)—C segments are *anti* to each other and the amide O atom is *anti* to the H atoms attached to the adjacent C atoms. Further, the conformations of the N—H bonds in the amide fragments are *anti* to the *ortho*-methyl groups in the adjacent benzene rings. The complete molecule is generated by inversion symmetry. The dihedral angle between the benzene ring and the NH—C(O)—CH_2_ segment in the two halves of the mol­ecule is 62.1 (2)°. In the crystal, N—H⋯O inter­molecular hydrogen bonds link the mol­ecules into sheet-like infinite chains along the *a* axis.

## Related literature

For our study of the effect of substituents on the structures of this class of compounds, see: Gowda *et al.* (2010**a* ▶,*b* ▶,c* ▶).
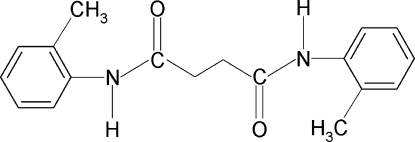


## Experimental

### 

#### Crystal data

C_18_H_20_N_2_O_2_
*M* *_r_* = 296.36
Monoclinic, 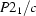

*a* = 11.586 (2) Å
*b* = 7.955 (1) Å
*c* = 8.803 (1) Å
β = 101.97 (2)°
*V* = 793.70 (19) Å^3^
*Z* = 2
Mo *K*α radiation
μ = 0.08 mm^−1^
*T* = 293 K
0.40 × 0.08 × 0.03 mm

#### Data collection

Oxford Diffraction Xcalibur diffractometer with a Sapphire CCD detector
Absorption correction: multi-scan (*CrysAlis RED*; Oxford Diffraction, 2009 ▶) *T*_min_ = 0.968, *T*_max_ = 0.998
3109 measured reflections
1615 independent reflections
987 reflections with *I* > 2σ(*I*)
*R*_int_ = 0.032

#### Refinement

*R*[*F* ^2^ > 2σ(*F* ^2^)] = 0.066
*wR*(*F* ^2^) = 0.163
*S* = 0.97
1615 reflections
104 parameters
1 restraint
H atoms treated by a mixture of independent and constrained refinement
Δρ_max_ = 0.19 e Å^−3^
Δρ_min_ = −0.21 e Å^−3^

### 

Data collection: *CrysAlis CCD* (Oxford Diffraction, 2009 ▶); cell refinement: *CrysAlis RED* (Oxford Diffraction, 2009 ▶); data reduction: *CrysAlis RED*; program(s) used to solve structure: *SHELXS97* (Sheldrick, 2008 ▶); program(s) used to refine structure: *SHELXL97* (Sheldrick, 2008 ▶); molecular graphics: *PLATON* (Spek, 2009 ▶); software used to prepare material for publication: *SHELXL97*.

## Supplementary Material

Crystal structure: contains datablocks I, global. DOI: 10.1107/S1600536811004442/ds2092sup1.cif

Structure factors: contains datablocks I. DOI: 10.1107/S1600536811004442/ds2092Isup2.hkl

Additional supplementary materials:  crystallographic information; 3D view; checkCIF report

## Figures and Tables

**Table 1:** Hydrogen-bond geometry (Å, °)

*D*—H⋯*A*	*D*—H	H⋯*A*	*D*⋯*A*	*D*—H⋯*A*
N1—H1*N*⋯O1^i^	0.85 (2)	1.99 (2)	2.840 (3)	173 (3)

Symmetry code: (i) 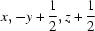.

**Fig. 1.**
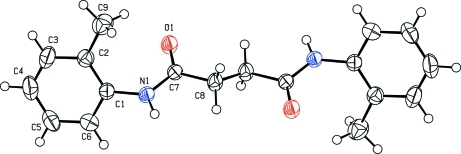
Molecular structure of (I), showing the atom labelling scheme and displacement ellipsoids are drawn at the 50% probability level.

**Fig. 2.**
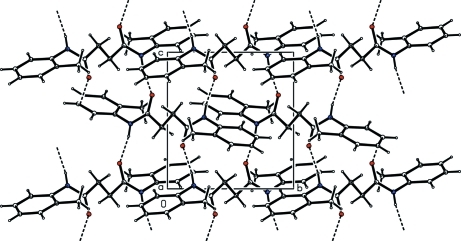
Molecular packing of (I) with hydrogen bonding shown as dashed lines.

## supplementary crystallographic information

### Comment

The amide moiety is an important constituent of many biologically important compounds. As a part of studying the substituent effects on the structures of this class of compounds (Gowda *et al.*, 2010**a*,*b*,c*), in the present work, the structure of *N*,*N*-Bis(2-methylphenyl)-succinamide has been determined (Fig.1). The conformations of N—H and C=O bonds in the C—NH—C(O)—C segments are *anti* to each other and the amide O atoms are *anti* to the H atoms attached to the adjacent C atoms. Further, conformations of the N—H bonds in the amide fragments are *anti* to the *ortho*-methyl groups in the adjacent benzene rings. The dihedral angle between the benzene ring and the NH—C(O)—CH_2_ segment in the two halves of the molecule is 62.1 (2)°.

Further, C1—N1—C7—C8 and C1a—N1a—C7a—C8a segments are nearly linear and so also the C1—N1—C7—O1 and C1a—N1a—C7a—O1a segments. The torsion angles of C2—C1—N1—C7 and C6—C1—N1—C7 are -64.0 (4)° and 117.6 (3)°.

The series of N—H···O intermolecular hydrogen bonds (Table 1) link the molecules into infinite chains (Fig. 2).

### Experimental

Succinic anhydride (0.01 mol) in toluene (25 ml) was treated drop wise with *o*-toluidine (0.01 mol) also in toluene (20 ml) with constant stirring. The resulting mixture was stirred for one hour and set aside for an additional hour at room temperature for completion of the reaction. The mixture was then treated with dilute hydrochloric acid to remove unreacted *o*-toluidine. The resultant *N*-(2-methylphenyl)succinamic acid was filtered under suction and washed thoroughly with water to remove the unreacted succinic anhydride and succinic acid. The compound was recrystallized to constant melting point from ethanol. The purity of the compound was checked by elemental analysis and characterized by its infrared and NMR spectra.

The *N*-(2-methylphenyl)succinamic acid obtained was then treated with phosphorous oxychloride and excess of *o*-toluidine at room temperature with constant stirring. The resultant mixture was stirred for 4 h, kept aside for additional 6 h for completion of the reaction and poured slowly into crushed ice with constant stirring. It was kept aside for a day. The resultant solid, *N*,*N*-Bis(2-methylphenyl)- succinamide was filtered under suction, washed thoroughly with water, dilute sodium hydroxide solution and finally with water. It was recrystallized to constant melting point from a mixture of acetone and chloroform. The purity of the compound was checked by elemental analysis, and characterized by its infrared and NMR spectra.

Needle like colorless single crystals used in the X-ray diffraction studies were were grown in a mixture of acetone and chloroform at room temperature.

### Refinement

The H atom of the NH group was located in a difference map and later restrained to the distance N—H = 0.86 (2) Å. The other H atoms were positioned with idealized geometry using a riding model with C—H = 0.93–0.97 Å. All H atoms were refined with isotropic displacement parameters (set to 1.2 times of the *U*_eq_ of the parent atom).

### Crystal data

**Table:**

C_18_H_20_N_2_O_2_	*F*(000) = 316
*M**_r_* = 296.36	*D*_x_ = 1.240 Mg m^−^^3^
Monoclinic, *P*2_1_/*c*	Mo *K*α radiation, λ = 0.71073 Å
Hall symbol: -P 2ybc	Cell parameters from 887 reflections
*a* = 11.586 (2) Å	θ = 2.6–27.6°
*b* = 7.955 (1) Å	µ = 0.08 mm^−^^1^
*c* = 8.803 (1) Å	*T* = 293 K
β = 101.97 (2)°	Needle, colourless
*V* = 793.70 (19) Å^3^	0.40 × 0.08 × 0.03 mm
*Z* = 2	

### Data collection

**Table:**

Oxford Diffraction Xcalibur diffractometer with a Sapphire CCD detector	1615 independent reflections
Radiation source: fine-focus sealed tube	987 reflections with *I* > 2σ(*I*)
graphite	*R*_int_ = 0.032
Rotation method data acquisition using ω scans	θ_max_ = 26.4°, θ_min_ = 3.1°
Absorption correction: multi-scan (*CrysAlis RED*; Oxford Diffraction, 2009)	*h* = −14→13
*T*_min_ = 0.968, *T*_max_ = 0.998	*k* = −9→7
3109 measured reflections	*l* = −5→11

### Refinement

**Table:**

Refinement on *F*^2^	Primary atom site location: structure-invariant direct methods
Least-squares matrix: full	Secondary atom site location: difference Fourier map
*R*[*F*^2^ > 2σ(*F*^2^)] = 0.066	Hydrogen site location: inferred from neighbouring sites
*wR*(*F*^2^) = 0.163	H atoms treated by a mixture of independent and constrained refinement
*S* = 0.97	*w* = 1/[σ^2^(*F*_o_^2^) + (0.0688*P*)^2^ + 0.5489*P*] where *P* = (*F*_o_^2^ + 2*F*_c_^2^)/3
1615 reflections	(Δ/σ)_max_ = 0.001
104 parameters	Δρ_max_ = 0.19 e Å^−^^3^
1 restraint	Δρ_min_ = −0.21 e Å^−^^3^

### Special details

**Table:**

Experimental. CrysAlis RED (Oxford Diffraction, 2009) Empirical absorption correction using spherical harmonics, implemented in SCALE3 ABSPACK scaling algorithm.
Geometry. All e.s.d.'s (except the e.s.d. in the dihedral angle between two l.s. planes) are estimated using the full covariance matrix. The cell e.s.d.'s are taken into account individually in the estimation of e.s.d.'s in distances, angles and torsion angles; correlations between e.s.d.'s in cell parameters are only used when they are defined by crystal symmetry. An approximate (isotropic) treatment of cell e.s.d.'s is used for estimating e.s.d.'s involving l.s. planes.
Refinement. Refinement of *F*^2^ against ALL reflections. The weighted *R*-factor *wR* and goodness of fit *S* are based on *F*^2^, conventional *R*-factors *R* are based on *F*, with *F* set to zero for negative *F*^2^. The threshold expression of *F*^2^ > σ(*F*^2^) is used only for calculating *R*-factors(gt) *etc*. and is not relevant to the choice of reflections for refinement. *R*-factors based on *F*^2^ are statistically about twice as large as those based on *F*, and *R*- factors based on ALL data will be even larger.

### Fractional atomic coordinates and isotropic or equivalent isotropic displacement parameters (Å^2^)

**Table:**

	*x*	*y*	*z*	*U*_iso_*/*U*_eq_	
C1	0.2287 (2)	0.4159 (3)	0.4527 (3)	0.0332 (6)	
C2	0.3283 (2)	0.3765 (4)	0.3956 (3)	0.0375 (7)	
C3	0.3804 (3)	0.5058 (4)	0.3267 (4)	0.0509 (8)	
H3	0.4469	0.4828	0.2866	0.061*	
C4	0.3357 (3)	0.6671 (4)	0.3168 (4)	0.0572 (9)	
H4	0.3712	0.7505	0.2682	0.069*	
C5	0.2395 (3)	0.7053 (4)	0.3778 (4)	0.0544 (9)	
H5	0.2106	0.8147	0.3732	0.065*	
C6	0.1859 (3)	0.5800 (4)	0.4459 (4)	0.0468 (8)	
H6	0.1206	0.6051	0.4879	0.056*	
C7	0.1138 (2)	0.1563 (3)	0.4500 (3)	0.0299 (6)	
C8	0.0525 (2)	0.0450 (4)	0.5479 (3)	0.0356 (7)	
H8A	0.1082	−0.0375	0.6013	0.043*	
H8B	0.0266	0.1130	0.6259	0.043*	
C9	0.3816 (3)	0.2030 (4)	0.4074 (4)	0.0540 (9)	
H9A	0.3508	0.1415	0.3140	0.065*	
H9B	0.3622	0.1452	0.4946	0.065*	
H9C	0.4658	0.2118	0.4212	0.065*	
N1	0.1685 (2)	0.2917 (3)	0.5239 (2)	0.0355 (6)	
H1N	0.155 (2)	0.310 (3)	0.614 (2)	0.043*	
O1	0.11453 (17)	0.1214 (2)	0.31422 (19)	0.0414 (6)	

### Atomic displacement parameters (Å^2^)

**Table:**

	*U*^11^	*U*^22^	*U*^33^	*U*^12^	*U*^13^	*U*^23^
C1	0.0376 (14)	0.0357 (16)	0.0271 (13)	−0.0083 (12)	0.0085 (11)	−0.0046 (12)
C2	0.0355 (14)	0.0414 (17)	0.0352 (15)	−0.0055 (13)	0.0065 (12)	−0.0030 (13)
C3	0.0444 (17)	0.061 (2)	0.0507 (19)	−0.0170 (17)	0.0179 (15)	0.0004 (17)
C4	0.068 (2)	0.047 (2)	0.057 (2)	−0.0246 (17)	0.0158 (17)	0.0051 (17)
C5	0.070 (2)	0.0332 (18)	0.062 (2)	−0.0114 (16)	0.0176 (18)	−0.0029 (16)
C6	0.0522 (18)	0.0400 (18)	0.0511 (19)	−0.0041 (14)	0.0171 (15)	−0.0092 (15)
C7	0.0344 (14)	0.0312 (14)	0.0249 (13)	−0.0012 (12)	0.0078 (10)	0.0012 (12)
C8	0.0444 (16)	0.0378 (16)	0.0259 (14)	−0.0092 (12)	0.0105 (12)	0.0028 (12)
C9	0.0439 (17)	0.058 (2)	0.062 (2)	0.0057 (16)	0.0154 (15)	0.0040 (17)
N1	0.0465 (13)	0.0373 (13)	0.0262 (11)	−0.0094 (11)	0.0158 (10)	−0.0048 (10)
O1	0.0570 (13)	0.0437 (12)	0.0264 (10)	−0.0154 (10)	0.0157 (9)	−0.0039 (9)

### Geometric parameters (Å, °)

**Table:**

C1—C2	1.387 (4)	C6—H6	0.9300
C1—C6	1.393 (4)	C7—O1	1.229 (3)
C1—N1	1.427 (3)	C7—N1	1.347 (3)
C2—C3	1.393 (4)	C7—C8	1.512 (3)
C2—C9	1.507 (4)	C8—C8^i^	1.509 (5)
C3—C4	1.380 (5)	C8—H8A	0.9700
C3—H3	0.9300	C8—H8B	0.9700
C4—C5	1.368 (4)	C9—H9A	0.9600
C4—H4	0.9300	C9—H9B	0.9600
C5—C6	1.376 (4)	C9—H9C	0.9600
C5—H5	0.9300	N1—H1N	0.853 (17)
			
C2—C1—C6	120.8 (3)	O1—C7—N1	123.6 (2)
C2—C1—N1	121.5 (2)	O1—C7—C8	121.4 (2)
C6—C1—N1	117.7 (2)	N1—C7—C8	115.0 (2)
C1—C2—C3	117.3 (3)	C8^i^—C8—C7	112.3 (3)
C1—C2—C9	122.8 (2)	C8^i^—C8—H8A	109.2
C3—C2—C9	119.9 (3)	C7—C8—H8A	109.2
C4—C3—C2	121.6 (3)	C8^i^—C8—H8B	109.2
C4—C3—H3	119.2	C7—C8—H8B	109.2
C2—C3—H3	119.2	H8A—C8—H8B	107.9
C5—C4—C3	120.5 (3)	C2—C9—H9A	109.5
C5—C4—H4	119.8	C2—C9—H9B	109.5
C3—C4—H4	119.8	H9A—C9—H9B	109.5
C4—C5—C6	119.2 (3)	C2—C9—H9C	109.5
C4—C5—H5	120.4	H9A—C9—H9C	109.5
C6—C5—H5	120.4	H9B—C9—H9C	109.5
C5—C6—C1	120.6 (3)	C7—N1—C1	124.5 (2)
C5—C6—H6	119.7	C7—N1—H1N	115.1 (19)
C1—C6—H6	119.7	C1—N1—H1N	119.6 (19)
			
C6—C1—C2—C3	−2.3 (4)	C2—C1—C6—C5	2.1 (4)
N1—C1—C2—C3	179.4 (2)	N1—C1—C6—C5	−179.5 (3)
C6—C1—C2—C9	176.8 (3)	O1—C7—C8—C8^i^	−30.5 (4)
N1—C1—C2—C9	−1.5 (4)	N1—C7—C8—C8^i^	150.9 (3)
C1—C2—C3—C4	0.6 (4)	O1—C7—N1—C1	3.3 (4)
C9—C2—C3—C4	−178.6 (3)	C8—C7—N1—C1	−178.1 (2)
C2—C3—C4—C5	1.4 (5)	C2—C1—N1—C7	−64.0 (4)
C3—C4—C5—C6	−1.6 (5)	C6—C1—N1—C7	117.6 (3)
C4—C5—C6—C1	−0.1 (5)		

Symmetry codes: (i) −*x*, −*y*, −*z*+1.

### Hydrogen-bond geometry (Å, °)

**Table:**

*D*—H···*A*	*D*—H	H···*A*	*D*···*A*	*D*—H···*A*
N1—H1N···O1^ii^	0.85 (2)	1.99 (2)	2.840 (3)	173 (3)

Symmetry codes: (ii) *x*, −*y*+1/2, *z*+1/2.
